# A Histone-Like Protein of Mycobacteria Possesses Ferritin Superfamily Protein-Like Activity and Protects against DNA Damage by Fenton Reaction

**DOI:** 10.1371/journal.pone.0020985

**Published:** 2011-06-16

**Authors:** Masaki Takatsuka, Mayuko Osada-Oka, Eisuke F. Satoh, Kengo Kitadokoro, Yukiko Nishiuchi, Mamiko Niki, Masayasu Inoue, Kazuhiro Iwai, Tetsuo Arakawa, Yoshihiro Shimoji, Hisashi Ogura, Kazuo Kobayashi, Anura Rambukkana, Sohkichi Matsumoto

**Affiliations:** 1 Department of Bacteriology, Osaka City University Graduate School of Medicine, Abeno-ku, Osaka, Japan; 2 Department of Gastroenterology, Osaka City University Graduate School of Medicine, Abeno-ku, Osaka, Japan; 3 Department of Molecular Pathology, Osaka City University Graduate School of Medicine, Abeno-ku, Osaka, Japan; 4 Department of Biomolecular Engineering, Graduate School of Science and Technology, Kyoto Institute of Technology, Matsugasakigosyokaidou-cho, Sakyo-ku, Kyoto, Japan; 5 Toneyama Institute for Tuberculosis Research, Osaka City University Medical School, Toyonaka, Osaka, Japan; 6 Department of Biophysics and Biochemistry, Graduate School of Medicine and Cell Biology and Metabolism Group, Graduate School of Frontier Biosciences, Osaka University, Suita, Osaka, Japan; 7 National Institute of Animal Health, Tsukuba, Ibaraki, Japan; 8 Department of Virology, Osaka City University Graduate School of Medicine, Abeno-ku, Osaka, Japan; 9 Department of Immunology, National Institute of Infectious Diseases, Shinjuku-ku, Tokyo, Japan; 10 Centers for Regenerative Medicine, Neuroregeneration, and Infectious Diseases, University of Edinburgh, Edinburgh, Scotland, United Kingdom; Charité-University Medicine Berlin, Germany

## Abstract

Iron is an essential metal for living organisms but its level must be strictly controlled in cells, because ferrous ion induces toxicity by generating highly active reactive oxygen, hydroxyl radicals, through the Fenton reaction. In addition, ferric ion shows low solubility under physiological conditions. To overcome these obstacles living organisms possess Ferritin superfamily proteins that are distributed in all three domains of life: bacteria, archaea, and eukaryotes. These proteins minimize hydroxyl radical formation by ferroxidase activity that converts Fe^2+^ into Fe^3+^ and sequesters iron by storing it as a mineral inside a protein cage. In this study, we discovered that mycobacterial DNA-binding protein 1 (MDP1), a histone-like protein, has similar activity to ferritin superfamily proteins. MDP1 prevented the Fenton reaction and protects DNA by the ferroxidase activity. The *K*_m_ values of the ferroxidase activity by MDP1 of *Mycobacterium bovis* bacillus Calmette-Guérin (BCG-3007c), *Mycobacterium tuberculosis* (Rv2986c), and *Mycobacterium leprae* (ML1683; ML-LBP) were 0.292, 0.252, and 0.129 mM, respectively. Furthermore, one MDP1 molecule directly captured 81.4±19.1 iron atoms, suggesting the role of this protein in iron storage. This study describes for the first time a ferroxidase-iron storage protein outside of the ferritin superfamily proteins and the protective role of this bacterial protein from DNA damage.

## Introduction

Iron is an essential element for virtually all living organisms, and is convertible between the ferrous ion (Fe^2+^) and ferric ion (Fe^3+^) under physiological pH. Living organisms employ this feature of iron for the catalytic centers of several critical enzymes, such as ribonucleotide reductase for the synthesis of DNA substrates and cytochromes involved in electron transport in respiration.

However, iron levels must be strictly regulated as it is potentially toxic to cells. Fe^3+^ is stable but forms insoluble hydroxy-aquo complexes and its solubility is under 10^−18^ M in physiological conditions; bacterial multiplication requires approximately 10^−7^ M iron [1]. Fe^2+^ is a reductant and generates reactive oxygen species (ROS) and reactive nitrogen species (RNS), which damage most cell components, including DNA, membranes, and proteins. In particular, the hydroxyl radical (•OH) is known as most reactive species in ROS and toxic for the cells. The hydroxyl radical is generated via the non-enzymatic Fenton reaction:
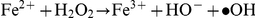



Most cells are equipped with mechanisms that protect the vital cellular components from ROS and NOS. The ferritin superfamily proteins, which are distributed among the three domains of life in both aerobic and anaerobic organisms, play a central role by detoxifying iron by converting Fe^2+^ to Fe^3+^ through its ferroxidase activity and subsequently store Fe^3+^ as a mineral. Ferritin superfamily proteins can be categorized by the presence or absence of heme. In bacteria, the former are called ferritin-like proteins or bacterioferritins and the latter are called mini-ferritins or DNA-binding protein in starved cells (Dps). Ferritin and bacterial ferritin-like proteins have exactly the same architecture, assembling in 24 subunits to form an inner nanocage, where a maximum of 4,500 Fe^3+^ can be sequestered as mineral after been oxidized from Fe^2+^ at the ferroxidase center [2], [3]. Dps forms the dodecameric oligomer and some of them possess DNA-binding activity. It similarly oxidizes Fe^2+^ at the ferroxidase center and stores a maximum 500 Fe^3+^ inside the protein [4]–[6].

*Mycobacterium tuberculosis* complex and *Mycobacterium leprae*, the etiologic agents of tuberculosis and leprosy respectively, are a serious threat to human health. According to WHO's weekly epidemiological report in 2010, tuberculosis alone kills 1.57 million people annually and 219,826 people are suffering from leprosy. These human pathogens are obligate intracellular bacteria that can survive or multiply particularly well in macrophages and Schwann cells. These invading mycobacteria restrict the host cell iron and iron-mediated ROS generation following NADPH dependent oxidative burst as efficient strategies against host defense [7]. Mycobacteria synthesize iron-chelating molecules, siderophores, and deprive the host cells of iron; they also produce antioxidant molecules, such as superoxide dismutase and catalase. Iron overload increases the risk of tuberculosis in the African human population [8] and a deficiency in siderophore synthesis prevents the replication of *M. tuberculosis* in macrophages [9]. Thus, coordination of iron homeostasis is essential to sustain survival and growth of mycobacteria in the host [10]. Both *M. tuberculosis* and *M. leprae* produce bacterioferritins [11]–[13], which should be involved controlling iron homeostasis of these pathogens.

DNA is an important cellular component and living organisms should be protected from damage caused by ROS. Because *M. tuberculosis* and *M. leprae* lack DNA-binding ferritin superfamily proteins like Dps [14], [15], it is thought that novel molecules that have not been identified mediate DNA protection in pathogenic mycobacteria. Colangeli et al have recently shown that mycobacterial histone-like protein Lsr2 protects DNA by acting as a physical shield [16]. However, Lsr2 lacks iron-binding and ferroxidase activities [16].

Mycobacteria including *M. tuberculosis* and *M. leprae* produce mycobacterial DNA-binding protein 1 (MDP1), also designated as laminin binding protein of *M. leprae* (ML-LBP) [17], [18] (Figure S1). The N- and C-terminal halves of this protein resemble bacterial histone-like proteins, IHF and HU and eukaryotic histone H1, respectively, and possesses MDP1/ML-LBP-specific DNA-binding motif (Figure S1) [17], [19]–[21]. In this study, we found that MDP1/ML-LBP has functions of a ferritin superfamily protein, that is both ferroxidase and iron-storage activities, and protects DNA not only physically but also prevents the iron-induced Fenton reaction. To our knowledge, this is the first report of a protein capable of storing and detoxifying iron other than ferritin superfamily proteins in living organisms.

## Results

### MDP1/ML-LBP possesses affinity for Fe^3+^ but not Fe^2+^

**Figure 1:**
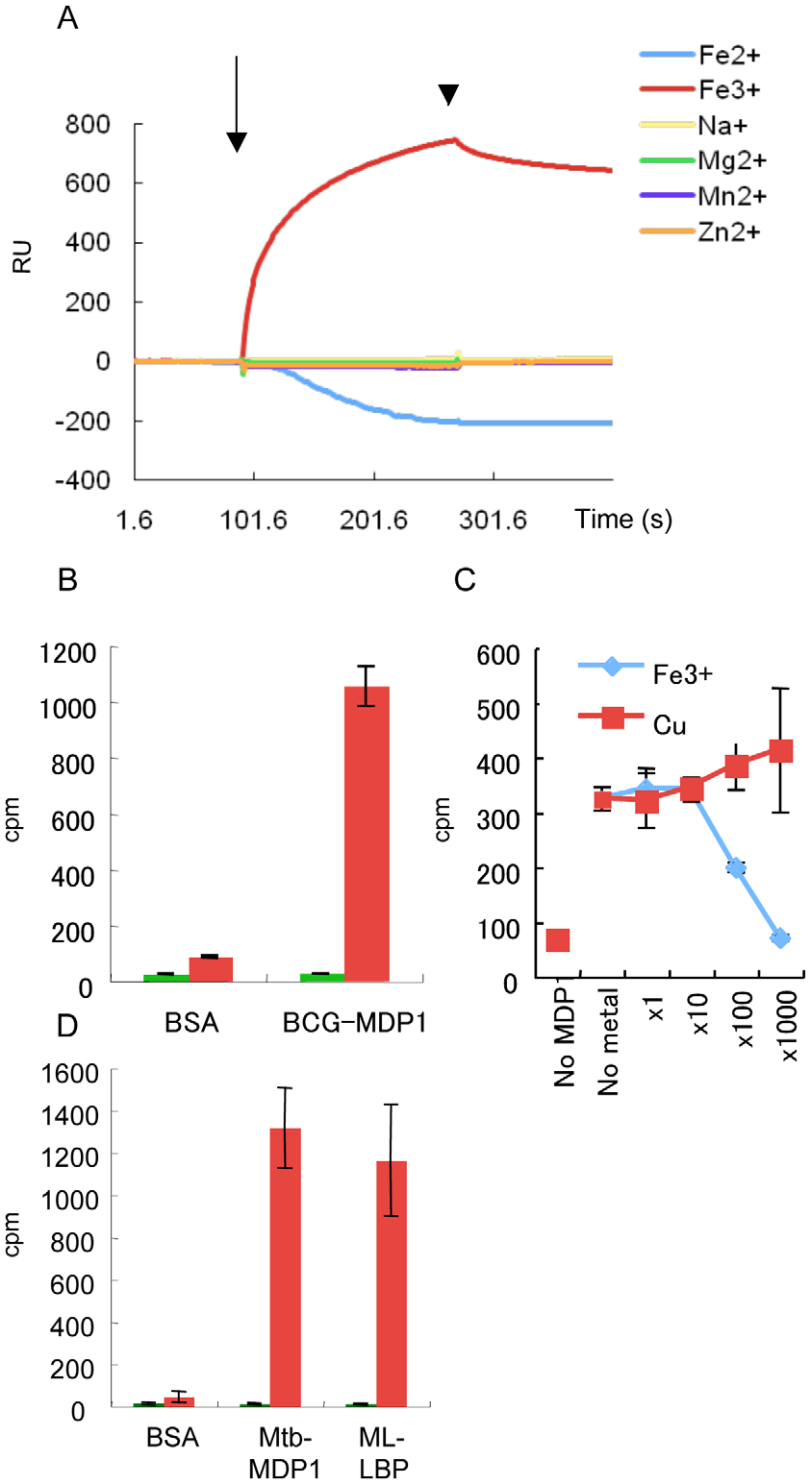
MDP1 binds to Fe^3+^. (A) SPR analysis by Biacore biosensor. Each metal (1 mM) was loaded into the sensor, where BCG-MDP1 was immobilized. The SPR responses (RU, resonance units) subtracted from the MDP1-free sensor are presented. Arrow, starting point of injection of metals. Arrow head, stopping point of metal injection. Horizontal axis, time (seconds). (B) Protein-^55^Fe interaction. Bovine serum albumin (BSA) or BCG-MDP1 was coated on a 96-well plastic plate. One µCi of ^55^FeCl_3_ was added to each well with (green bars) or without (red bars) incubation in 10 mM ascorbic acid. The level of bound iron was counted using a scintillation counter. Vertical axis, cpm (counts per minutes) of ^55^Fe. (C) Inhibition of MDP1-^55^Fe interaction by cold Fe^3+^. One µCi of ^55^FeCl_3_ was added to the BCG-MDP1 coated or non-coated wells in the presence or absence of various molar concentrations of Fe^3+^ or Cu^2+^ as indicated. Vertical axis, level of bound ^55^Fe. (D) Protein-^55^Fe interaction. BSA, Mtb-MDP1, or ML-LBP was coated on a 96-well plastic plate. One µCi of ^55^FeCl_3_ was added to each well with (green bars) or without (red bars) incubation in 10 mM ascorbic acid. Levels of bound iron were counted using a scintillation counter.

MDP1/ML-LBP mediates several cellular processes through binding to DNA, sugar-containing molecules, and proteins both inside and outside of mycobacteria [19], [21]–[23]. Therefore we analyzed the potential interaction between MDP1 of *Mycobacterium bovis* bacillus Calmette Guérin (BCG) (BCG-MDP1) and each ligand (analyte) by measuring surface plasmon resonance (SPR) with a BIAcore 2000 biosensor. In the course of this study, we unexpectedly observed strong increase of SPR, suggesting interaction between the analyte and immobilized ligand, when Fe^3+^ (ammonium iron (III) citrate) alone was added to the BCG-MDP1 immobilized sensor (Fig. 1A). By contrast, a gradual decrease of SPR was detected when Fe^2+^ (FeSO_4_) was injected. Such obvious increase and decrease of SPR were not detected on injection of other metals, such as Cu^2+^, Mg^2+^, Mn^2+^, and Zn^2+^ (Fig. 1A).

Resonance units (RU) analyzed by BIAevaluation software of SPR are tightly correlated with the weight of bound protein ligand. However RU are not always correlated with weight in the case of protein-small molecule interaction [24]. In addition, it was reported that protein-metal interaction changes the protein structure, which in turn causes the changes of SPR [25]. Therefore, we next examined the binding capacity of BCG-MDP1 to Fe^3+^ by using radioactive ^55^Fe. The results showed that ^55^Fe bound to BCG-MDP1 but not to BSA coated on the ELISA plate (Fig. 1B). By contrast, iron pre-incubated with 10 mM ascorbic acid, which reduces Fe^3+^ to Fe^2+^, did not interact with BCG-MDP1. In addition, the BCG-MDP1-^55^Fe interaction was inhibited in the presence of excess amounts of cold Fe^3+^ but not Cu^2+^ (CuSO_4_) (Fig. 1C). Taken together, these data demonstrate that BCG-MDP1 binds Fe^3+^.

In order to determine whether the close mycobacterial MDP1-homologues have similar binding activity, we cloned the gene encoding MDP1 from *M. tuberculosis* H37Rv and purified it as a recombinant histidine-tagged protein (Mtb-MDP1) after expression in *E. coli*. Similarly, we also obtained recombinant ML-LBP of *M. leprae* after purification from *E. coli* expressing ML-LBP as described previously [18]. We analyzed the ^55^Fe binding activities of these recombinant proteins. Like BCG-MDP1, both Mtb-MDP1 and ML-LBP bound to Fe^3+^ but not Fe^2+^ (Fig. 1D).

We next examined how many iron atoms MDP1 chelates using ICP-MS. BCG-MDP1 retained in the heparin column was incubated in the presence of 1 mM Fe^3+^ solution for 30 min and unbound iron was extensively washed. The BCG-MDP1-iron complex was eluted by 2 M NaCl and the iron content in the solution was determined by ICP-MS. The calculated data showed that one BCG-MDP1 contained 81.4±19.1 iron atoms (Table 1).

**Table 1:** Iron contents determined by ICP-MS.

Samples	(µg/kg)	Iron atoms/protein
BCG-MDP1	90.0	0.6
Buffer alone	155.0	ND
Iron-loaded BCG-MDP1	10,660.0±2505.6	81.4±19.1
Iron-loaded Mtb-MDP1	18,166.7±138.7	138.7±35.5

ND, not detected.

### MDP1/ML-LBP has ferroxidase activity

The SPR analysis showed reduction of RU when Fe^2+^ was injected into a BCG-MDP1-immobilized sensor (Fig. 1A), implying some unknown responses of MDP1 in the presence of Fe^2+^. Although MDP1 has no motifs for ferroxidase, we next examined whether MDP1/ML-LBP has enzymatic ferroxidase bioactivity.

**Figure 2:**
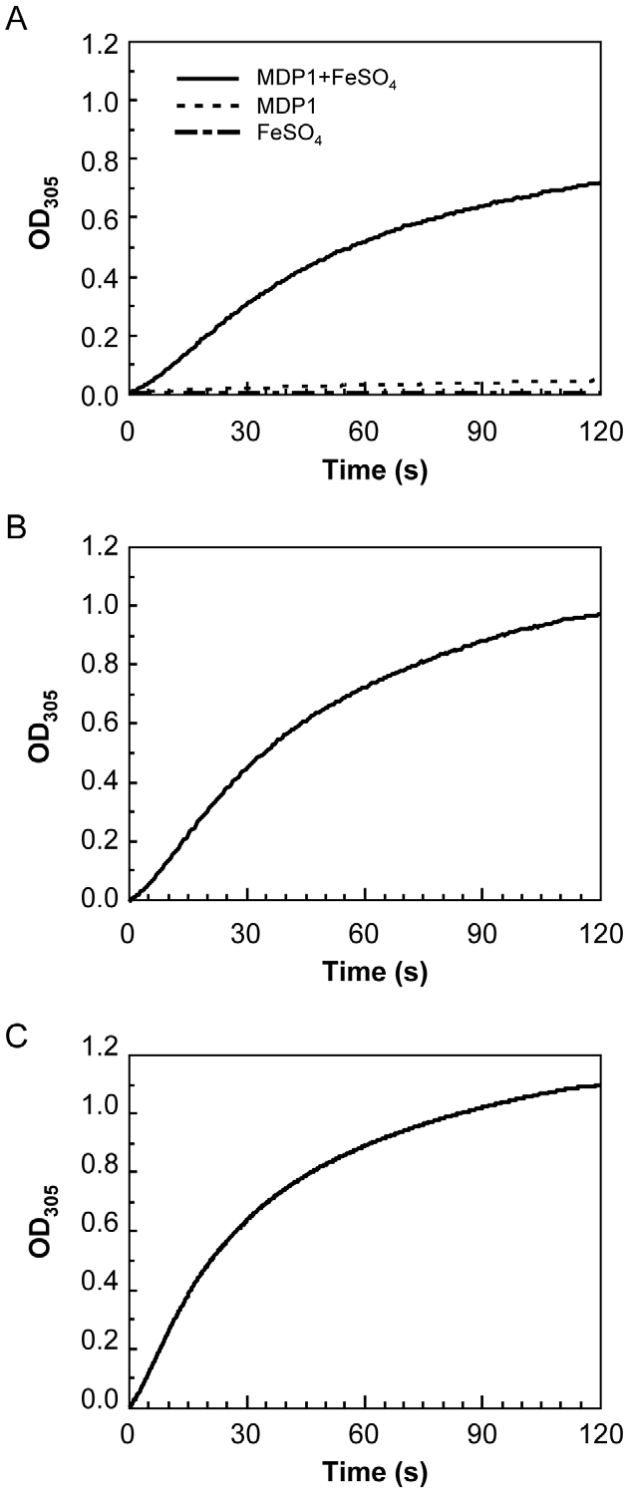
Ferroxidase activity of MDP1/ML-LBP. The conversion from Fe^2+^ to Fe^3+^ was determined by measuring the absorbance at 305 nm. (A) broken line, 1.4 µM BCG-MDP1; dush-dotted line, 0.4 mM FeSO_4_; solid line, 0.4 mM FeSO_4_+1.4 µM BCG-MDP1. (B) solid line, 0.4 mM FeSO_4_+1.4 µM Mtb-MDP1. (C) solid line, 0.4 mM FeSO_4_+1.4 µM ML-LBP. The reaction was monitored for 120 seconds.

**Figure 3:**
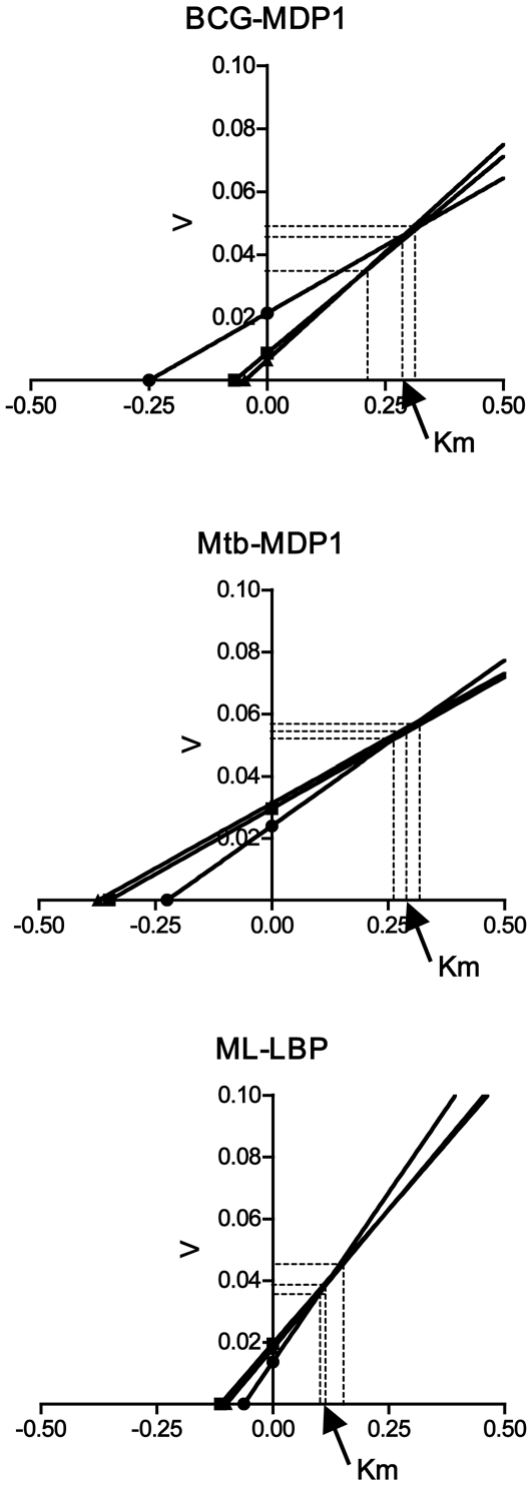
Ferroxidase activity of BCG-MDP1, Mtb-MDP1, and ML-LBP. The conversion from Fe^2+^ to Fe^3+^ was monitored by spectral analysis at 305 nm. 0.0125–0.1 mM FeSO_4_ were added to 1.4 µM BCG-MDP1 (A), Mtb-MDP1 (B) and ML-LBP (C). Direct linear plots were shown by the straight lines intercepting X-axis with −1/*K*_m_. *K*_m_ are taken as the median value from each series.

We performed spectral analysis at 305 nm, because ferroxidase activity is the reaction, which converts Fe^2+^ to Fe^3+^, resulting in production of the dinuclear iron (μ-oxo-bridged Fe^3+^ dimmers). To analyze the activity, protein at a concentration of 1.4 µM was incubated at 37°C for 120 seconds in 20 mM Tris-HCl (pH 7.0). Both BCG-MDP1 and buffer alone (0.4 mM FeSO_4_) did not absorb at 305 nm during 120 seconds incubation (Fig. 2A, broken line and dush-dotted line, respectively). In contrast, in the presence of both BCG-MDP1 and 0.4 mM FeSO_4_ there was a rapid increase in the absorbance (Fig. 2A, solid line) with a *K*_m_ value of 0.292 mM (Fig. 3A). Similarly, the enzymatic activity of both Mtb-MDP1 and ML-LBP possess identical ferroxidase activities (Fig. 2B and 2C), of which *K*_m_ values were calculated as 0.252 mM and 0.129 mM, respectively (Fig. 3B and C). By contrast, BSA and bovine histone H1 did not show such an increase in absorbance at 305 nm (Figure S2).

### MDP1/ML-LBP prevents the iron-induced Fenton reaction

**Figure 4:**
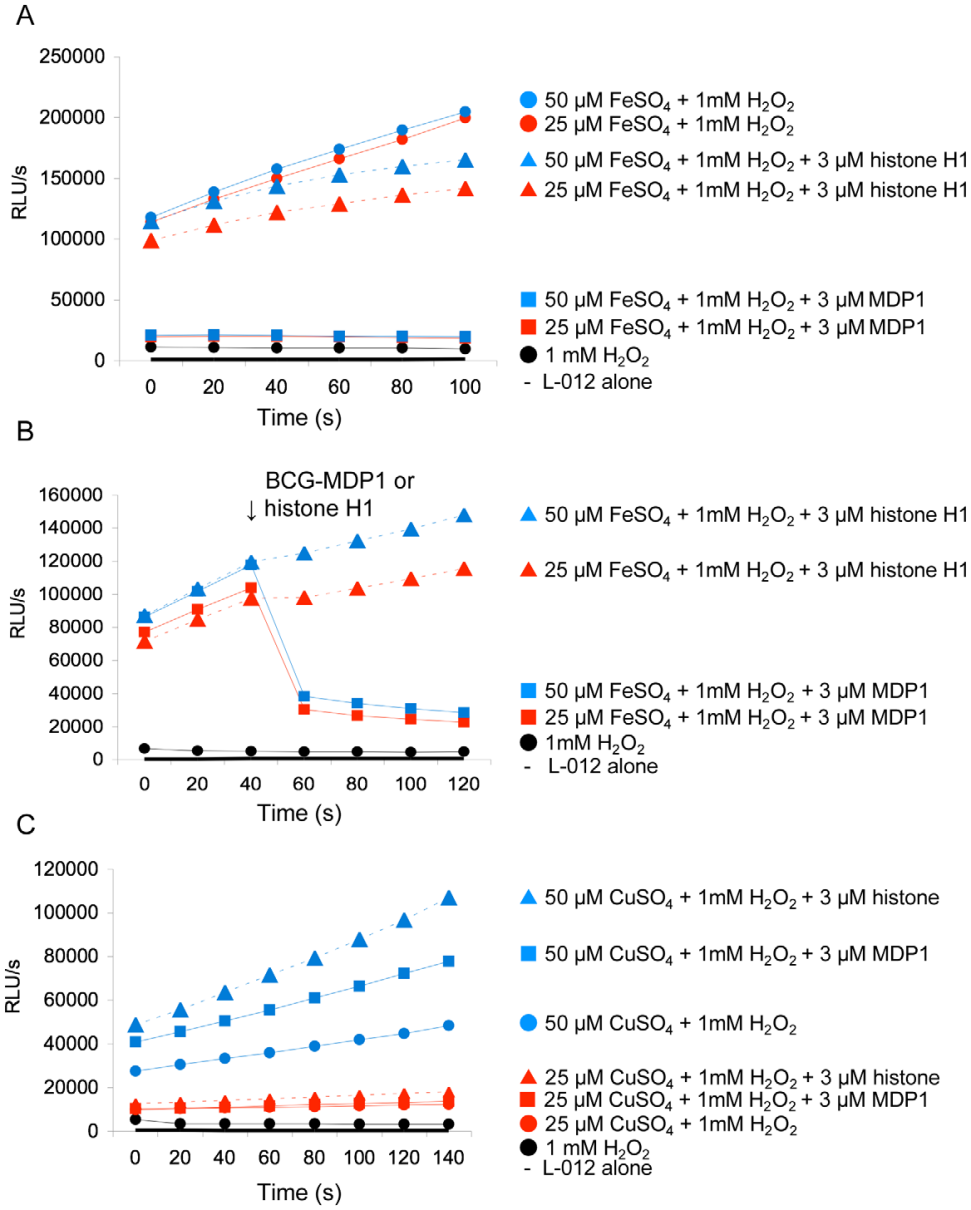
MDP1 suppresses the generation of the hydroxyl radical by the Fenton reaction. The hydroxyl radical level was measured by a L-012 probe sensitive to oxygen radicals. L-012 probe was added in all samples. RLU, relative luminescence units. (A) Proteins were added at the initiating time point of the Fenton reaction by H_2_O_2_ and FeSO_4_. (B) Proteins were added 40 seconds after initiating the Fenton reaction. (C) The effect of proteins on the Fenton like reaction generated by H_2_O_2_ and CuSO_4_.

**Figure 5:**
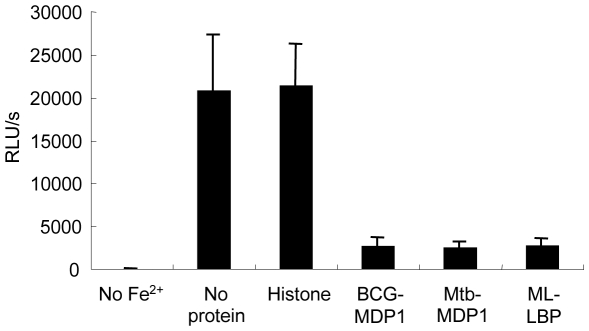
Mtb-MDP1 and ML-LBP suppress the generation of the hydroxyl radical by the Fenton reaction. The level of hydroxyl radical produced by the Fenton reaction was monitored using L-012, a probe that is sensitive to reactive oxygen, in the presence or absence of 3 µM proteins, such as bovine histone H1 (Histone), BCG-MDP1, Mtb-MDP1, and ML-LBP. The level of hydroxyl radical 10 sec after initiation of the reaction is shown. The data is representative of 3 independent experiments.

Since ferroxidase activity can prevent the Fenton reaction by converting Fe^2+^ into Fe^3+^, we examined whether MDP1 and ML-LBP could abolish the Fenton reaction. Hydroxyl radical generated in the presence of 1 mM H_2_O_2_ and 25 or 50 µM FeSO_4_ by the Fenton reaction was monitored by “8-amino-5-chloro-7-phenylpyrido[3,4-d]pyridazine-1,4-(2H,3H) dione” (L-012), which reacts with reactive oxygen species and develops strong chemiluminescence (CHL) [26]. Hydroxyl radical was generated depending on the concentration of Fe^2+^ (Fig. 4A). In this experimental setting, the addition of 3 µM BCG-MDP1 remarkably suppressed the generation of hydroxyl radical (Fig. 4A). Furthermore, the addition of BCG-MDP1 after initiation of the Fenton reaction suppressed the production of hydroxyl radical even under optimal generating conditions (Fig. 4B). Similarly, both Mtb-MDP1 and ML-LBP suppressed the generation of hydroxyl radical by the Fenton reaction (Fig. 5). In contrast, neither histone H1 nor 3 µM BSA suppressed the Fenton reaction (Fig. 4A & B). Furthermore, the suppressive activity of 3 µM Mtb-MDP1 or ML-LBP was equivalent to 30 µM of the antioxidant sugar, ascorbic acid (data not shown).

Hydroxyl radical can be generated in the presence of bivalent metals other than iron and H_2_O_2_. In order to clarify whether the suppressive effect on the Fenton reaction by MDP1 is dependent on its ferroxidase activity, we studied the effects of BCG-MDP1 on Cu^2+^-dependent Fenton-like reactions. For these studies hydroxyl radical was generated in the presence of 25 or 50 µM CuSO_4_ and 1 mM H_2_O_2_ (Fig. 4C). The data showed that neither MDP1 nor histone H1 suppressed Cu^2+^-induced Fenton-like reactions, but in fact enhanced the reaction. These data reinforce the hypothesis that inhibition of iron-induced Fenton reactions by MDP1 is indeed dependent on ferroxidase activity.

### Dual mechanism of DNA protection by MDP1/ML-LBP

**Figure 6:**
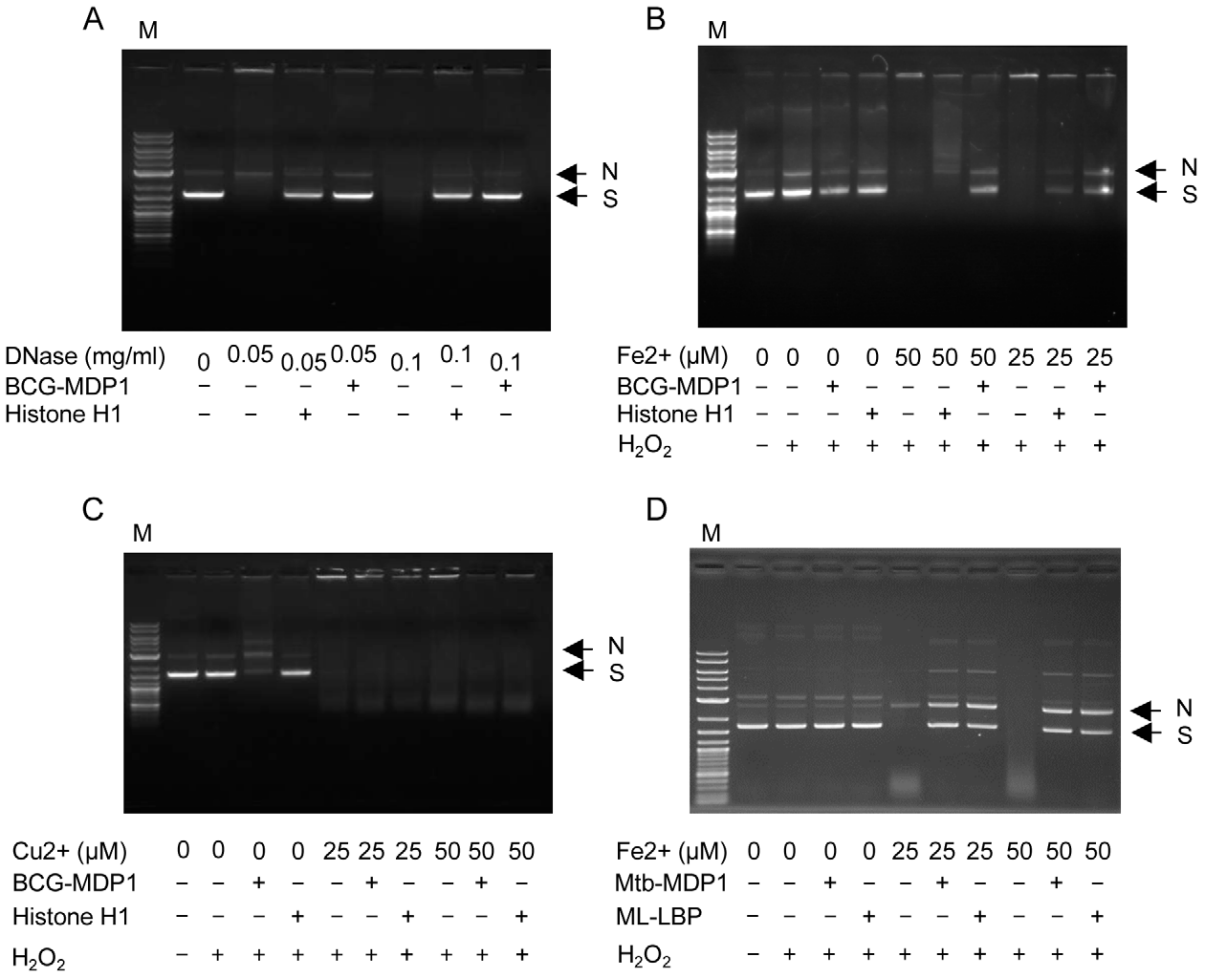
DNA protective activity of MDP1/ML-LBP. The plasmid DNA (pUC19) was treated with DNase 1 (A), H_2_O_2_ and FeSO_4_ (Fenton reaction, B and C), and H_2_O_2_ and CuSO_4_ (Fenton-like reaction, D) in the presence or absence of proteins, such as histone H1, BCG- MDP1, Mtb-MDP1, and ML-LBP. After reaction for 30 min, DNA was extracted, fractionated with gel electrophoresis and visualized by UV light after staining with ethidium bromide. M, marker. N, nicked DNA. S, supercoiled DNA.

We next examined the effect of MDP1 on the protection of DNA. First, plasmid DNA (pUC19) was incubated with DNase1 in the presence or absence of 3 µM BCG-MDP1 and histone H1. We found that DNA was protected equally by MDP1 and histone H1 (Fig. 6A). Thus, both MDP1 and histone H1 protect DNA from DNase1 by acting as physical shields.

Comparison of the effects of MDP1 and histone H1 on DNA damage induced by the Fenton reaction revealed that DNA was degraded in the presence of 25 or 50 µM FeSO_4_ and 1 mM H_2_O_2_ (Fig. 6B). BCG-MDP1 protected DNA from degradation by the Fenton reaction, whereas the level of protection exerted by histone H1 was comparatively lower (Fig. 6B). By contrast, neither BCG-MDP1 nor histone H1 could protect DNA from Cu^2+^-induced Fenton-like reactions (Fig. 6C), further suggesting that MDP1 protects DNA by suppressing iron-induced Fenton reactions mediated through its ferroxidase activity. As expected both Mtb-MDP1 and ML-LBP also protected DNA from digestion with DNase1 and the damage caused by the Fenton reaction in a similar manner to BCG-MDP1 (Fig. 6D). Taken together, these data suggest that MDP1 and ML-LBP protect DNA in a dual fashion, by acting as a physical barrier and minimizing the Fenton reaction.

### The role of MDP1 in the detoxication of iron in *Mycobacterium*

**Figure 7:**
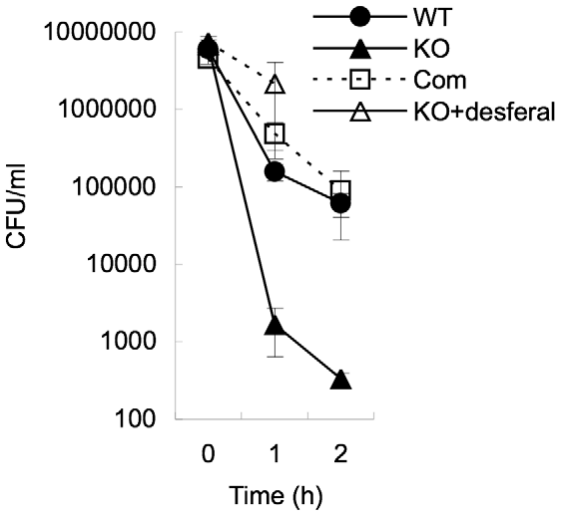
MDP1-deficient *M. smegmatis* is susceptible to H_2_O_2_ treatment. Bacteria at an exponential growth phase were treated with 12.5 mM H_2_O_2_ and the survival rate was determined by CFU. Closed circle, the wild-type *M. smegmatis* (WT). Closed triangle, MDP1-deficient *M. smegmatis* (KO), Open square, Ms-MDP1-complemented strain (Com). Open triangle, the MDP1-deficient strain pretreated with 40 mM desferal to confirm that iron is involved in the killing by H_2_O_2_ treatment (KO+desferal).

Finally, we assessed the role of MDP1 in the detoxication of iron in *Mycobacterium* itself. MDP1 is presumed to be essential for slow growers of mycobacteria, such as BCG and *M. tuberculosis* [27], [28] and cannot be knocked out. Therefore we employed the MDP1-homologue (Ms-MDP1)-deficient strain of rapid grower *Mycobacterium smegmatis* [29]. We detected the ferroxidase activity of purified Ms-MDP1, of which the *K*_m_ value was 0.136 mM (Figure S2). We compared the resistance to H_2_O_2_ among wild type and mutant strains of *M. smegmatis*, including Ms-MDP1-deficient, and Ms-MDP1-complemented strains. These strains were treated with 12.5 mM H_2_O_2_ in the logarithmic phase of growth and the survival rate was determined by a colony forming unit (CFU) assay. There was a 100-fold reduction in survival of the Ms-MDP1-deficient strain (Fig. 7). We also pretreated bacteria with 40 mM desferal, an iron chelator. This treatment remarkably inhibited the bactericidal effect of H_2_O_2_ (Fig. 7), showing that major bactericidal effect with H_2_O_2_ treatment is depending on the Fenton reaction. Together, our data show that MDP1 play a role in iron detoxfication and bacterial survival.

### Phylogenetic analysis of MDP1/ML-LBP homologues and ferritin-superfamily proteins

**Figure 8:**
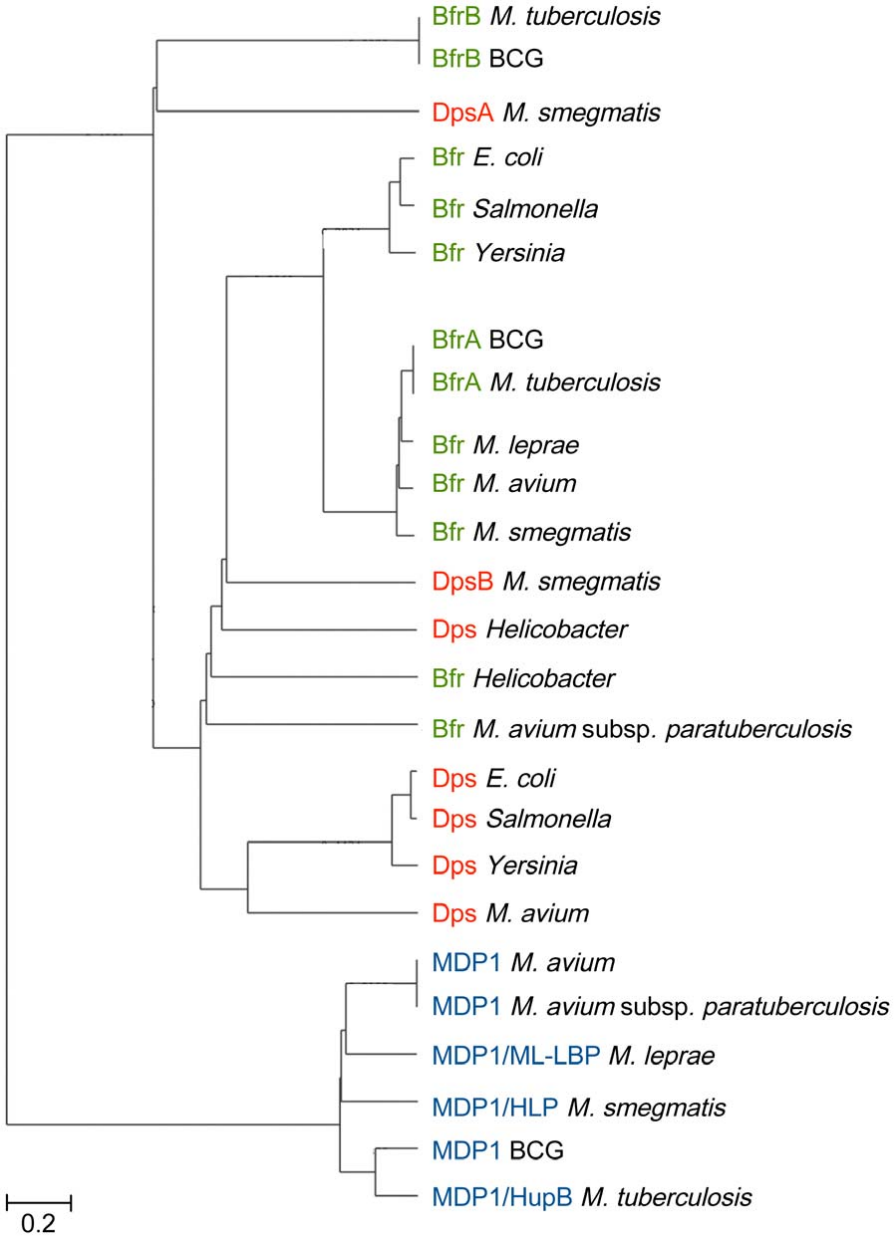
Phylogenetic tree of MDP1/ML-LBP homologues and ferritin superfamily proteins. The amino acid sequences of BCG-MDP1 (AB013441), *M. tuberculosis*-MDP1 (NP_217502), *M. avium*-MDP1 (YP_883003), *M. avium* subsp. *paratuberculosis* -MDP1 (NP_961958), *M. smegmatis*-MDP1 (YP_886729), *M. leprae*-ML-LBP (MDP1) (NP_302157), BCG-bacterioferritin A (BfrA) (YP_978002), BCG-BfrB (YP_979983), *M. tuberculosis*-BfrA (CAB10050), *M. tuberculosis*-BfrB (AAF06357), *M. avium*-Bfr (YP_882020), *M. avium* subsp. *paratuberculosis* (P45430), *M. smegmatis* Bfr (ABK70328), *M. leprae*-Bfr (AAA21339), *E. coli*-Bfr (AP_004454), *Salmonella enterica*-Bfr (CBY97651), *Yersinia pestis*-Bfr (AAS60481), *Helicobacter pylori*-Bfr (CAA06826), *M. avium*-Dps (YP_884099), *M. avium* subsp. *paratuberculosis*-Dps (NP_962494), *M. smegmatis*-DpsA (ABK75435), *M. smegmatis*-DpsB (ABK69831), *E. coli*-Dps (U00096.2), *Salmonella enterica*-Dps (Car32361.1), *Yersinia pestis*-Dps (AL590842.1), and *Helicobacter pylori*-Dps (1JI4_L) are compared by using Genetyx (Ver.16.1) software according to the UPGMA algorism. Values, 0.2, in the figure represent evolutionary distances.

We accomplished molecular evolutionary analysis to recognize the phylogenetic diversity of MDP1/ML-LBP homologues and ferritin-superfamily proteins. We constructed phylogenetic dendrogram of MDP1/ML-LBP homologues and ferritin-superfamily proteins (bacterioferritin and Dps) of mycobacteria, such as BCG, *M. tuberculosis*, *Mycobacterium avium*, *Mycobacterium avium* subsp. *paratuberculosis*, *M. smegmatis*, and *M. leprae*. We also applied amino acid sequences of other bacterial ferritin-superfamily proteins of *E. coli*, *Salmonella enterica*, *Yersinia pestis*, and *Helicobacter pylori* and the dendrogram was created using clustering with the Unweighted Pair Group Method with Arithmetic Mean (UPGMA) by GENETYX software (Genetyx, Tokyo, Japan). In the phylogenetic tree (Figure 8), MDP1/ML-LBP homologues formed different cluster from ferritin-superfamily proteins, supporting phylogenetic distinctiveness between MDP1/ML-LBP homologues and ferritin superfamily proteins.

## Discussion

Iron in living organisms is a double-edged sword, as it is an essential and beneficial element but is also harmful. Therefore, living organisms strictly control iron homeostasis. The ferritin superfamily proteins are distributed across all three domains of life and contribute to iron detoxication through oxidation and storage as Fe^3+^, the nontoxic form of iron. Bacteria produce two types of ferritin superfamily proteins, one is bacterioferritin and another is Dps (also called miniferritin). Bacterioferritin is distributed among mycobacteria [12], [13], [30]–[32] but pathogenic mycobacteria, such as *M. tuberculosis* complex and *M leprae*, lack DNA-binding ferritin superfamily proteins like Dps. Here we show a histone-like mycobacterial protein, MDP1/ML-LBP has similar activity with ferritin superfamily proteins and protect DNA by preventing the Fenton reaction.

MDP1/ML-LBP is a major cellular component of mycobacterial cell [17], [18] and is a multifunctional molecule depending on interaction with biomolecules, such as DNA, laminin [18], [33], glycosaminoglycans [19], [33], and glycolipids [22], [28], and in turn controls gene expression, infection, and cell wall integrity [19], [22], [23], [28], [33]. In contrast, in this study, we identified enzymatic activity of MDP1 itself that should be involved in iron-homeostasis of mycobacteria.

This study was initiated by the unexpected detection of the increase and decrease of SPR, when Fe^3+^ and Fe^2+^ were loaded into BCG-MDP1-immobilized sensors, respectively (Fig. 1A). Because altered protein structure changes RU of SPR [25], we examined whether MDP1 actually binds to iron. We have shown that BCG-MDP1 binds to Fe^3+^, but not to Fe^2+^ (Fig. 1B and C) and captures 81.4±19.1 iron atoms/protein, as measured by ICP-MS (Table 1). We also confirmed similar iron-binding activity of recombinant Mtb-MDP1 and ML-LBP (Fig. 1D). MDP1 is eluted in the 150–210 kDa fraction by gel filtration [17], suggesting that 7–10 MDP1 subunits oligomerize. Thus, it is estimated that oligomerized MDP1 can sequester 532–760 iron molecules, which is comparable to the iron-storage activity of ferritin superfamily proteins. The reduction of RU of SPR, when Fe^2+^ was injected into MDP1-immobilized sensor might be due to structural change of MDP1.

MDP1/ML-LBP is phylogenetically distinctive protein from ferritin superfamily proteins as shown in Figure 8. A remarkable difference in the method of iron storage between MDP1 and ferritin is that MDP1 directly captures Fe^3+^, while ferritin incorporates iron after oxidizing Fe^2+^ using oxygen or hydrogen peroxide at the ferroxidase center. Thus, ferroxidase activity is dispensable for iron sequestration by MDP1/ML-LBP but not by ferritin superfamily proteins. These data suggest that MDP1/ML-LBP is a new type of iron detoxication and storage protein. The ferroxidase activity of MDP1 using O_2_ as oxidant was slightly slower but comparable with that of apo-horse ferritin (*K*_m_ value, 0.2127 mM) as shown in Figure S3. Taken together, to our knowledge, this is the first report of a molecule possessing iron oxidation and storage other than ferritin superfamily proteins.

The amino acid sequence of MDP1/ML-LBP resembles histones in eukaryotes and histone-like proteins in bacteria, thus it probably has a similar molecular root with those proteins. It is intriguing how MDP1/ML-LBP has evolved to acquire similar activity as ferritin superfamily proteins during evolution.

Mycobacteria are successful pathogens, producing various kinds of effector molecules that facilitate the long-term survival in the host. Acquisition of iron and preventing iron-dependent generation of ROS are essential events for the replication and survival in the host for the pathogens [9], [34], [35]. Dps is involved in the survival of bacterial pathogens, such as *Listeria* and *Salmonella*, by detoxifying iron [36]. Here we showed that Ms-MDP1 plays a critical role in iron detoxication in *M. smegmatis*, which produces Dps (Fig. 7). Therefore, it is reasonable to consider that MDP1/ML-LBP has more significant roles in the live and virulence of pathogenic mycobacteria, which lack Dps. That may be one of reasons why MDP1 is essential in *M. tuberculosis* [27]. Recent study conducted by Kumar et al, showing protection of DNA by MDP1 from DNase 1 and the Fenton reaction, also support our finding [37]. Our finding on the new type of iron detoxication and storage protein will provide important information on the strategy and insight into virulence of mycobacteria.

## Methods

### Bacterial strains and proteins

MDP1-deficient *Mycobacterium smegmatis* was a gift from Dr. Thomas Dick [29]. The complemented strain was generated previously [22]. BCG-MDP1 and *M. smegmatis*-MDP1 (Ms-MDP1 or HLP) was purified from cultured BCG Tokyo and *M. smegmatis* mc^2^155, respectively, on Sauton media according to the method described previously [17]. Bovine histone H1 and bovine serum albumin (BSA) were purchased from Roche and Sigma, respectively. The open reading flame of MDP1 was amplified from the *M. tuberculosis* H37Rv genome using the following primers, forward: 5′-ccc cat atg aac aaa gca gag ctc att gac-3′, reverse: 5′-ccc aag ctt ttt gcg acc ccg ccg agc gg-3′, containing the restriction sites of NdeI and HindIII. The 645 bp amplicon was cloned into pCR2.1-TOPO (Invitrogen, Carlsbad, CA) and further subcloned as an NdeI-HindIII fragment in the corresponding site of pET-22b (+) (Novagen, Darmstadt, Germany). The plasmid was transformed and expressed in the *Escherichia coli* BL-21 strain and Mtb-MDP1 (Rv2986c) was purified using Ni-NTA agarose (Qiagen, Valencia, CA) with 300 mM imidazole in phosphate buffer (pH 7.4) after obtaining acid soluble proteins [17]. The *M. leprae*-MDP1 (ML-LBP or ML1683) was purified in the same way utilizing a previously constructed expression vector [18]. Concentrations of proteins were determined by Bradford's method [38] using BSA as a standard.

### SPR analysis by Biacore biosensor

The interaction between BCG-MDP1 and metal was monitored by measuring SPR using a BIAcore 2000 biosensor (GE Healthcare, Buckinghamshire, UK). All binding reactions were performed at 25°C in 10 mM HEPES buffer, pH 7.4, including 150 mM NaCl, 1% BSA, 3 mM EDTA, and 0.005% surfactant P20 (HBSEP buffer). BCG-MDP1 was immobilized to the dextran matrix on the CM5 sensor chip (GE) using an amine coupling kit according to the manufacturer's instructions (GE). Metals, such as ammonium iron (III) citrate, CuSO_4_, MgSO_4_, MnCl_2_, ZnSO_4_, and FeSO_4_, were dissolved in HBSEP buffer to a final metal ion concentration of 1 mM and injected over both the control and BCG-MDP1-immobilized sensor chip.

### Detection of MDP1-^55^Fe interaction

A 96-well ELISA plate (Sumitomo, Tokyo, Japan) was coated overnight with 5 µg/ml BCG-MDP1, Mtb-MDP1, ML-LBP or BSA in borate-buffered saline (pH 9.2) at 4°C. The wells were then washed with phosphate buffered saline (PBS). One hundred µl of PBS containing 1 µCi of ^55^FeCl_3_ (PerkinElmer, Boston, MA) was added into the wells coated with the proteins. In some cases, ^55^FeCl_3_ was incubated in 10 mM ascorbic acid for 30 min before addition to the wells. After 30 min incubation, the wells were washed four times in PBS containing 0.05% Tween 20. Then pre-warmed water at 37°C containing 1% SDS and 10 mM cold ammonium iron (III) citrate was added and the wells were flushed by pipetting. Twenty µl of the suspension was then spotted onto Whatman 3 MM paper and its radioactivity was counted by a liquid scintillation counter (Aloka, Tokyo, Japan).

### Inductively coupled plasma mass spectrometry (ICP-MS) analysis

After a heparin column was washed out with 10 mM sodium phosphate (pH 7), 1 ml of 0.1 mg/ml BCG-MDP1 or Mtb-MDP1 solution in pure water was incubated for 30 min. After washing, the column was incubated in the presence of 1 mM ammonium iron (III) (1 ml) citrate for 30 min. Unbound iron was washed out and the MDP1-iron complex was eluted by sodium phosphate buffer containing 2 M NaCl. Each sample of approximately 0.1 g was weighed accurately in quartz plates, and they were gradually carbonized with 0.5 ml of sulfuric acid using hot plates. They were then heated for 8 hours at 510°C in an electric furnace and finally washed. After cooling, the residue was dissolved with 1.25 ml of nitric acid and diluted to 25 ml with ultra pure water (the analysis solution). The Fe concentration of each analysis solution was determined by ICP-MS (Model ELAN DRC II; Perkin Elmer). We repeated the experiments 5 and 3 times for BCG-MDP1 and Mtb-MDP1, respectively.

### Assay of ferroxidase activity

To determine whether MDP1 converts Fe^2+^ to Fe^3+^ using O_2_ as oxidant, 0.4 mM FeSO_4_ as 15 mM solution at pH 3.5 were added to 1.4 mM MDP1 in 20 mM Tris-HCl (pH 7.0) including with 150 mM NaCl. The protein solution was pre-incubated at 37°C for 10 min. UV spectral absorption at 305 nm has been traditionally used to monitor a μ-oxo-bridged Fe^3+^ dimmers, which was monitored by spectrophotometer U-3000 (HITACHI, Tokyo, Japan). MDP1 produced no UV absorbance in the absence of Fe^2+^. Lineweaver-Burk plot was used for considering the inverse values of the absorption per 60 s after addition of 0.0125–0.1 mM FeSO_4_. The Kinetic parameters (*K*_m_) were calculated by direct linear plot through the two points, *K*_m_ and V that satisfy the Michaelis-Menten equation exactly for every observation [39]. The best estimates, *K*_m_ was taken as the medians of the two sets of estimates.

### Monitoring hydroxyl radical generation

Level of hydroxyl radical generation by the Fenton reaction were measured using L-012 (0.08 mM) as a probe as previously described [26]. The total reaction volume was 50 µl containing 20 mM Tris HCl (pH 7.5), 50 mM NaCl. Three µM Histone H1, BCG-MDP1, Mtb-MDP1, and ML-LBP were incubated with 25 or 50 µM FeSO_4_ and 1 mM H_2_O_2_. After addition of L-012 (5 µl), CHL intensity was recorded continuously for 20–140 seconds using a Luminescence Reader BLR-201 (Aloka, Tokyo, Japan). The level of hydroxyl radical generation by the combination of 25 or 50 µM CuSO_4_ and 1 mM H_2_O_2_ was monitored in the same way.

### DNA protection assay

DNA protection from oxidative damage and enzymatic digestion was assessed *in vitro* using pUC19 plasmid DNA (2686 bp, 50 nM), purified by a Qiaprep spin plasmid miniprep kit (Qiagen). The total reaction volume was 20 µl in pure water. Plasmid DNA was allowed to interact with MDP1, ML-LBP, or bovine Histone H1 for 30 min prior to the introduction of 25 or 50 µM FeSO_4_ and 1 mM H_2_O_2_ or treatment with 50 or 100 µg/ml DNase I (Sigma, St.Louis, MO). The reaction mixtures were incubated for 30 min at room temperature before the reactions were stopped by incubation with 1% SDS and proteins were degraded by treatment with 20 µg/ml PronaseK (Sigma) for 30 min. Then the plasmid was extracted using phenol-chloroform extraction and 10 µl of sample was loaded on 1% agarose gel in TAE buffer for electrophoresis. The gel was stained with ethidium bromide and DNA was visualized under UV light.

### H_2_O_2_ treatment

*M. smegmatis* strains were grown to an OD600 of 0.5 to 0.7 in LB medium at 37°C. The bacterial culture was then diluted to 0.1 OD by the medium for the assay. The reaction was initiated by adding a final concentration of 12.5 mM H_2_O_2_ and incubated for 0, 1, or 2 hours. If necessary, bacteria were pretreated with 40 mM desferal (Sigma) for 5 min before exposure to H_2_O_2_. At each time point, the bacterial suspension was serially diluted using sterilized water and plated onto LB agar (Sigma). Living bacteria were enumerated by counting CFU after incubation at 37°C for 4–6 days.

## Supporting Information

Figure S1 MDP1/ML-LBP homologues. Blast search was performed for amino acid sequence of BCG-MDP1 against all protein database using the National Center for Biotechnology Information's (NCBI) BLAST server and proteins with over 150 of total score are aligned. Conserved domain of Integration host factor (IHF) and HU were shown by yellow box, which was identified with domain search using NCBI server. MDP1-specific DNA binding region [20] that interacts with GC-rich DNA was indicated by red.
(DOC)

Figure S2 Ferroxidase activity of Ms-MDP1. The conversion from Fe^2+^ to Fe^3+^ was scanned by measuring the absorbance at 305 nm. Black line, 0.4 mM FeSO_4_; blue line, 0.4 mM FeSO_4_+1.4 µM Histone H1; red line, 0.4 mM FeSO_4_+1.4 µM Ms-MDP1.
(TIF)

Figure S3 Ferroxidase activity of Mtb-MDP1 and ferritin using hydroxyl peroxide, as the oxidant The conversion from Fe^2+^ to Fe^3+^ was monitored by spectral analysis at 305 nm. Hydroxyl peroxide (6–108 mM) were added to the solution of either 0.5 µM Mtb-MDP1 (A) or horse ferritin (B) including with 21 µM FeSO_4_.
(TIF)
