# Supplementary material for: A Histone-Like Protein of Mycobacteria Possesses Ferritin Superfamily Protein-Like Activity and Protects against DNA Damage by Fenton Reaction
Source: PLoS One. 2011 Jun 16;6(6):e20985. doi: 10.1371/journal.pone.0020985 (PMC3116847; doi:10.1371/journal.pone.0020985)
Supplement: Figure S1 — MDP1/ML-LBP homologues. Blast search was performed for amino acid sequence of BCG-MDP1 against all protein database using the National Center for Biotechnology Information's (NCBI) BLAST server and proteins with over 150 of total score are aligned. Conserved domain of Integration host factor (IHF) and HU were shown by yellow box, which was identified with domain search using NCBI server. MDP1-specific DNA binding region [20] that interacts with GC-rich DNA was indicated by red. (DOC) [file pone.0020985.s001.doc]

Accession Total

Numbers score Bacterial species (strains)

AB013441 1 -----MNKAELIDVLTQKLGSDRRQATAAVENVVDTIVRAVHKGDSVTITGFGVFEQRRRAARVARNPRTGETVKVKPTS 75 370 *Mycobacterium bovis* (BCG)

CAB46493 1 -----MNKAELIDVLTQKLGSDRRQATAAVENVVDTIVRAVHKGDSVTITGFGVFEQRRRAARVARNPRTGETVKVKPTS 75 368 *Mycobacterium bovis*

ZP_07428191 1 -----MNKAELIDVLTQKLGSDRRQATAAVENVVDTIVRAVHKGDSVTITGFGVFEQRRRAARVARNPRTGETVKVKPTS 75 223 *Mycobacterium tuberculosis* (SUMu004)

ZP_05773801 1 -----MNKAELIDVLTQKLGSDRRQATAAVENVVDTIVRAVHKGDSVTITGFGVFEQRRRAARVARNPRTGETVKVKPTS 75 204 *Mycobacterium tuberculosis* (K85)

YP_001850032 1 -----MNKAELIDVLTQKLGSDRRQATAAVENVVDTIVRAVHKGDSVTITGFGVFEQRRRAARVARNPRTGETVKVKPTS 75 201 *Mycobacterium marinum*

AAX89120 1 -----MNKAELIDVLTQKLGSDRRQATAAVENVVDTIVRAVHKGDSVTITGFGVFEQRRRAARVARNPRTGETVKVKPTS 75 201 *Mycobacterium ulcerans*

NP_217502 1 -----MNKAELIDVLTQKLGSDRRQATAAVENVVDTIVRAVHKGDSVTITGFGVFEQRRRAARVARNPRTGETVKVKPTS 75 201 *Mycobacterium tuberculosis* (H37Rv)

ZP_04747612 1 -----MNKAELIDVLTQKLGSDRRQATAAVENVVDTIVRAVHKGDSVTITGFGVFEQRRRAARVARNPRTGETVKVKPTS 75 199 *Mycobacterium kansasii*

NP_302157 1 -----MNKAELIDVLTQKLGSDRRQATAAVENVVDTIVRAVHKGDSVTITGFGVFEQRRRAARVARNPRTGETVKVKPTS 75 197 *Mycobacterium leprae*

ZP_06852690 1 -----MNKAELIDVLTQKLNTDRRQATAAVENVVDTIVRAVHKGDSVTITGFGVFEQRRRAARVARNPRTGETVKVKPTS 75 195 *Mycobacterium parascrofulaceum*

YP_952958 1 -----MNKAELIDVLTEKLGSDRRQATAAVENVVDTIVRAVHKGESVTITGFGVFEQRRRAARVARNPRTGETVKVKPTS 75 195 *Mycobacterium vanbbalenii*

YP_883003 1 MSEGLMNKAELIDVLTQKLNTDRRQATAAVENVVDTIVRAVHKGDSVTITGFGVFEQRRRAARVARNPRTGETVKVKPTS 80 193 *Mycobacterium avium* (104)

YP_001135482 1 -----MNKAELIDVLTEKLGSDRRQATAAVENVVDTIVRAVHKGESVTITGFGVFEQRRRAARVARNPRTGETVKVKPTS 75 193 *Mycobacterium gilvum*

NP_961958 1 -----MNKAELIDVLTQKLNTDRRQATAAVENVVDTIVRAVHKGDSVTITGFGVFEQRRRAARVARNPRTGETVKVKPTS 75 193 *Mycobacterium avium* subsp. paratuberculosis

YP_886729 1 -----MNKAELIDVLTTKMGTDRRQATAAVENVVDTIVRAVHKGDSVTITGFGVFEQRRRAARVARNPRTGETVKVKPTS 75 190 *Mycobacterium smegmatis*

YP_120423 1 -----MNKAELIDVLTEKLGTDRRTATAAVEHVVDTIVRAVHKGQSVTITGFGVFEQRKRAPRVARNPRTGETVKVKATS 75 189 *Nocardia farcinica*

YP_001704022 1 -----MNKAELIDVLTQKLGSDRRQATAAVEHVVDTIVRTVHKGESVTITGFGVFEQRRRAARVARNPRTGETVKVKPTS 75 187 *Mycobacterium abscessus*

YP_003647783 1 -----MNKAELIEELTEKLGTDRRTAGAAVEAIVDTIVRAVHAGDSVTITGFGVFEQRKRAARVARNPRTGETVKVKPTS 75 185 *Tsukamurella paurometabola*

YP_639088 1 -----MNKAELIDALTTKMGTDRRQATEAVENVVDTIVRAVHKGDSVTITGFGVFEQRRRAARVARNPRTGETVKVKPTS 75 184 *Mycobacterium* sp. (MCS)

ZP_04387154 1 -----MNKAELIDVLTEKLGSDRRTATDAVEHVVDTIVRAVHRGESVTITGFGVFEQRRRAARVARNPRTGETVKVKPTS 75 179 *Rhodococcus erythropolis*

YP_002783744 1 -----MNKAELIDVLTEKLGTDRRTATEAVEHVVDTIVRAVHRGDSVTITGFGVFEQRRRAARVARNPRTGETVKVKPTS 75 179 *Rhodococcus opacus*

YP_706432 1 -----MNKAELIDVLTEKLGTDRRTATEAVEHVVDTIVRAVHRGDSVTITGFGVFEQRRRAARVARNPRTGETVKVKPTS 75 179 *Rhodococcus jostii*

YP_003274311 1 -----MNKAELIDELTKKLDADRKTATEAVELIVDTIVRAVNKGESVTITGFGVFEKRRRAPRVARNPRTGETVKVRATS 75 175 *Gordonia bronchialis*

YP_004007817 1 -----MNKAELIDVLTEKLGTDRRTASEAVEHVVDTIVRAVHAGESVTITGFGVFEQRRRAARVARNPRTGETVKVKPTS 75 173 *Rhodococcus equi*

ZP_05224111 1 -------------MLTQKLNTDRRQATAAVENVVDTIVRAVHKGDSVTITGFGVFEQRRRAARVARNPRTGETVKVKPTS 67 173 *Mycobacterium intracellulare*

BAA78330 76 VPAFRPGAQFKAVVSGAQRLPAEGPAVKRGV-GA----SAAKK-VAKKAPAKKATKAA---------------------- 127

CAB46493 76 VPAFRPGAQFKAVVSGAQRLPAEGPAVKRGV-GA----SAAKK-VAKKAPAKKATKAA---------------------- 127

ZP_07428191 76 VPAFRPGAQFKAVVSGAQRLPAEGPAVKRGVGAS---------------------------------------------- 109

ZP_05773801 76 VPAFRPGAQFKAVVSGAQRLPAEGPAVKRGV-GA----SAAKK-VAKKAPAKKATKAAKKAA-------TKAPA------ 136

YP_001850032 76 VPAFRPGAQFKAVVSGAQRLPAEGPAVKRGVMAS----AAAKK-AAKKAP-------AKKAA-------TKTAAKKAATK 136

AAX89120 76 VPAFRPGAQFKAVVSGAQRLPAEGPAVKRGVMAS----AAAKK-AAKKAP-------AKKAA-------TKTAA------ 130

NP_217502 76 VPAFRPGAQFKAVVSGAQRLPAEGPAVKRGV-GA----SAAKK-VAKKAPAKKATKAAKKAA-------TKAPA------ 136

ZP_04747612 76 VPAFRPGAQFKAVVAGAQKLPAEGPAVKRGV-GT----SAAKK-AAKKAP-------ARKAA-------TKAPAKKAATK 135

NP_302157 76 VPAFRPGAQFKAVVAGAQRLPLEGPAVKRGVATS-----AAKKAAIKKAP-------VKKAL------------------ 125

ZP_06852690 76 VPAFRPGAQFKAVVSGAQRLPSEGPAVKRGVVAS----GAAKKTAAKKAP-------AKKAA-------AK--------- 128

YP_952958 76 VPAFRPGAQFKAVVSGAQKLPAEGPAVKRGVTAT----STARK-AAKKAP-------AKKAA-------VK--------- 127

YP_883003 81 VPAFRPGAQFKAVVSGAQRLPSEGPAVKRGVVGG-----AAKKTAAKKAP-------AKKAA-------AK--------- 132

YP_001135482 76 VPAFRPGAQFKAVVSGAQKLPAEGPAVKRGVAAA----STARK-AAKKAP-------AKKAAP------AK--------- 128

NP_961958 76 VPAFRPGAQFKAVVSGAQRLPSEGPAVKRGVVGG-----AAKKTAAKKAP-------AKKAA-------AK--------- 127

YP_886729 76 VPAFRPGAQFKAVISGAQKLPADGPAVKRGVTAG-----PAKK-AAKKAP-------AKKAA-------AK--------- 126

YP_120423 76 VPAFRPGAQFKAVIAGKQKLAATGPAVKRGVNAP--------------VA-------AKKTAAKKTTAAAK--------- 125

YP_001704022 76 VPTFRPGAQFKAVVSGAQKLPADGPAVKRGSTA---------------AP-------AKRAA-------AK--------- 117

YP_003647783 76 VPAFRPGAQFKAVISGAAKLPASGPAVRRSSATATPTKAAKKT-AAKKAP-------AKKAAP------AK--------- 132

YP_639088 76 VPAFRPGAQFKAIVSGAQKLASEGPAVKRGVAAG-----------PAKRA-------AKKAPAKKT--AAK--------- 126

ZP_04387154 76 VPAFRPGAQFKAVIAGGQKLPATGPAVKRGV--A--------------AP-------------------AT--------- 111

YP_002783744 76 VPAFRPGAQFKAVIAGGQKLPATGPAVKRGAAAP-----------ATKAA-------AKKAAAKKT--AAK--------- 126

YP_706432 76 VPAFRPGAQFKAVIAGGQKLPATGPAVKRGAAAP-----------ATKAA-------AKKAAAKKT--AAK--------- 126

YP_003274311 76 VPAFRPGAQFKAVVAGKQKLAATGPAVKRGSG-------------ATSAP-------AKKAAP------AK--------- 120

YP_004007817 76 VPAFRPGAQFKALIAGGQKLPSSGPAVKRGAAEP------------VKKA-------AKKTAAKKT--AAK--------- 125

ZP_05224111 68 VPAFRPGAQFKAVVSGAQRLPSDGPAVKRGVVGGT---GAAKKTAAKKAP-------AKKAA-------AK--------- 121

BAA78330 128 ---KKA----ATKAP---AK-----KA---------ATKAPAKKAV-KATKSPAKKV-TK----A-VKKTAVK------- 169

CAB46493 128 ---KKA----ATKAP---AK-----KA---------ATKAPAKKAV-KATKSPAKKV-TK----A-VKKTAVK------- 169

ZP_07428191 --------------------------------------------------------------------------------

ZP_05773801 137 ---KKA----ATKAP---AK-----KA---------ATKAPAKKAV-KATKSPAKKV-TK----A-VKKTAVK------- 178

YP_001850032 137 APAKKA----ATKAP---AK-----KA---------ATKAPAKKAVTKVTKAPAKKV-TK----ATVKKTAAK------- 183

AAX89120 131 ---KKA----ATKAP---AK-----KA---------ATKAPAKKAVTKVTKAPAKKV-TK----ATVKKTAAK------- 174

NP_217502 137 ---RKA----ATKAP---AK-----KA---------ATKAPAKKAV-KATKSPAKKV-TK----A-VKKTAVK------- 178

ZP_04747612 136 APAKKA----ATKAP---AK-----TA---------A-KAPAKKAATKATKAPAK-A-TK----TTAKKAAAK------- 180

NP_302157 126 --AKKA----ATKAP---AK-----KA---------V-KAPAKKIT-TAVKVPAKKA-TK-----VVKKVAAK------- 167

ZP_06852690 129 ---KTA----AKKAP---AK-----KA---------ATKAPAKKAA--TK-APAKKAATK----APVKKAATK------- 170

YP_952958 128 ---KAA---PAKKAP---AK-----KA---------A---PAKKA------A-VKKA-------APAKKAPAK------- 160

YP_883003 133 ---KAP----AKKAA---AK------------------KAPAKKAA--VKKAPARKAATK----APVRKAATK------- 171

YP_001135482 129 ---KTA---AKKAAP---AK-----KA---------ATKAPAKKA------APAKKA-------APAKKTAAK------- 165

NP_961958 128 ---KAP----AKKAA---AK------------------KAPAKKAA--VKKAPARKAATK----APVRKAATK------- 166

YP_886729 127 ---KTATKAAAKKAP---AK-----KA---------ATKAPAKKAA--TK-APAKKAATK----APAKKAATK------- 172

YP_120423 126 ---KT---AAKKTTT---ATKAPAKKT--TATK-APAKSTARKTT------TATKTAAKK--APA-KKT-TATKASATKA 183

YP_001704022 118 ---KAA---PAKKAP---AK-----KA---------A---PAKKA------P-VKKA-------VVKKAAPVK------- 150

YP_003647783 133 ---KA---AVKKAAP---AK-----KA---A----PAKKAVVKKA------APAKKATPA-------KK-AVT------- 170

YP_639088 127 ---KTTA-AAKKTAP--------AKKSTAAAKKTAPAKKTAAKKTT----AAAKKTAPAKKSTAAAKKT-----APA--- 182

ZP_04387154 112 ---KAA---AKKAAA---KK-----TA---------AKKAPAKK-------APAKTA---------AKKTVAT------- 145

YP_002783744 127 ---KT---AAKKA----PAKTAAAKKT--VAKKVAPAKTAAAKKT------VAKKVAPAK--TAAAKKT-AAKKAPA--- 182

YP_706432 127 ---KT---AAKKAA---PAKTTAAKKT--AAKK-APAKTTAAKKT------VAKKVAPAK--TAA-KKT-AAKKAPA--- 181

YP_003274311 121 ---KT---AAKKAAP---AK-----KT---AAKKAPAKTTAAKKA------APAKKTAAK-------KA-AAK------- 162

YP_004007817 126 ---KT---AAKKTAAKAPAKTVA-KKT--AAKA--PAKTVA-KKT------AAK--APAK--TAA-KKT-AAK-APA--- 177

ZP_05224111 122 ---KAP----AKKAA---AK------------------KAPAKKAA--VKKAPAKKAAVKK---APARKAATK------- 161

BAA78330 170 -----ASV----------RKA-----------------------ATK----APAKKA-AAK---------------RPAT 191

CAB46493 170 -----ASV----------RKA-----------------------ATK----APAKKA-AAK---------------RPAT 191

ZP_07428191 --------------------------------------------------------------------------------

ZP_05773801 179 -----ASV----------RKA-----------------------ATK----APAKKA-AAK---------------RPAT 200

YP_001850032 184 -----APV----------RKA-----------------------ATK----APAKKA-AAK---------------RPAT 205

AAX89120 175 -----APV----------RKA-----------------------ATK----APAKKA-AAK---------------RPAT 196

NP_217502 179 -----ASV----------RKA-----------------------ATK----APAKKA-AAK---------------RPAT 200

ZP_04747612 181 -----APV----------RKA-----------------------ATK----APAKKA-AAK---------------RPAT 202

NP_302157 168 -----APV----------RKA-----------------------TTR----ALAKKA-AVK------------------- 185

ZP_06852690 171 -----APVKKAAA-----KKA-----------------------ATK----APVKKA-AAK---------------RPAS 197

YP_952958 161 ---KAAPA----------KKA-AVKKAAP---------------AKK----APAKKA-AVKKAPAK-----KAA---PAK 198

YP_883003 172 -----APAKKVAAKKAPAKKA-----------------------ATK----APAKKA-ASK---------------APAR 203

YP_001135482 166 ---KAAPA----------KKAPAAKKAAP---------------AKK----APAKKA-ATKAAPAK-----KA----PAK 203

NP_961958 167 -----APAKKVAAKKAPAKKA-----------------------ATK----APAKKA-ASK---------------APAR 198

YP_886729 173 -----APA----------KKA-----------------------AAK----APAKKA-ATK---------------APAK 194

YP_120423 184 SATKATAA----------KKTTTAAKKT---------TATKATAAKKT---TAAKKAPAKKTA---------------AK 226

YP_001704022 151 ---K-APV----------KKA-VVKKAAP---------------VKKAVTKAPAKKA-ATK-APAK-----KAATKAPAK 193

YP_003647783 171 ---KAAPA----------KKAPAKKTVTK---------AAP---AKK----APAKKAPAKKAAPAKKAPAKKAATKAPAK 221

YP_639088 183 ---KKAAT----------K--------------APAKKAA----AKA----PAKKATAAKKTA----------------S 211

ZP_04387154 146 ---RLPPR----------RLR---PRPQP---------------RRR----LPPRLP-PRRLRPR----------PQPRR 179

YP_002783744 183 ---KTAAA----------KKTVAKKVA-------PAKTAA----AKK----TAAKKAPAKT-A---------------AK 218

YP_706432 182 ---KTTAA----------KKTVAKKVA-------PAKTA-----AKK----TAAKKAPAKTTA---------------AK 217

YP_003274311 163 ---KATAA----------KKAPAKK-------------AAP---AKK----TAAKKAPAKRTRR---------------- 193

YP_004007817 178 ---KT-AA----------KKTVAKKTATKAPAKAPAKTAAKKTVAKK----TAAK-APAKTAAK-----------KTVAK 227

ZP_05224111 162 -----APAKKTAAKKAPARKA-----------------------ATK----APAKKA-ASK---------------STAR 193

BAA78330 192 KAPAKKATA-RRGRK- 205

CAB46493 192 KAPAKKAAA-RRGRK- 205

ZP_07428191 ----------------

ZP_05773801 201 KAPAKKATA-RRGRK- 214

YP_001850032 206 KAPAKKATSTRRGRK- 220

AAX89120 197 KAPAKKATSTRRGRK- 211

NP_217502 201 KAPAKKATA-RRGRK- 214

ZP_04747612 203 KAPAKKATSTRRGRK- 217

NP_302157 186 KAPAKKVTAAKRGRK- 200

ZP_06852690 198 KAPAKKATA-RRGRR- 211

YP_952958 199 KAPAKKAPA-KRGRK- 212

YP_883003 204 KAAAKKTTA-RRGRK- 217

YP_001135482 204 KAPAKKAPA-KRGRK- 217

NP_961958 199 KAAAKKTTA-RRGRK- 212

YP_886729 195 KAAAKKAPA-KKGRR- 208

YP_120423 227 KTAARR---------- 232

YP_001704022 194 KAPAKKAPA-KKGRK- 207

YP_003647783 222 KAPAKKAPA-KRGKK- 235

YP_639088 212 KAPARKAPA-KKGRK- 225

ZP_04387154 180 RLPP------------ 183

YP_002783744 219 KTAAKKAPA-KRAK-- 231

YP_706432 218 KTAAKKAPA-KRAK-- 230

YP_003274311 ----------------

YP_004007817 228 KTAAKKAPA-KRTARK 242

ZP_05224111 194 KAPAKKTTA-RRGRR- 207
