# Supplementary figures and images for: A Histone-Like Protein of Mycobacteria Possesses Ferritin Superfamily Protein-Like Activity and Protects against DNA Damage by Fenton Reaction
Source: PLoS One. 2011 Jun 16;6(6):e20985. doi: 10.1371/journal.pone.0020985 (PMC3116847; doi:10.1371/journal.pone.0020985)

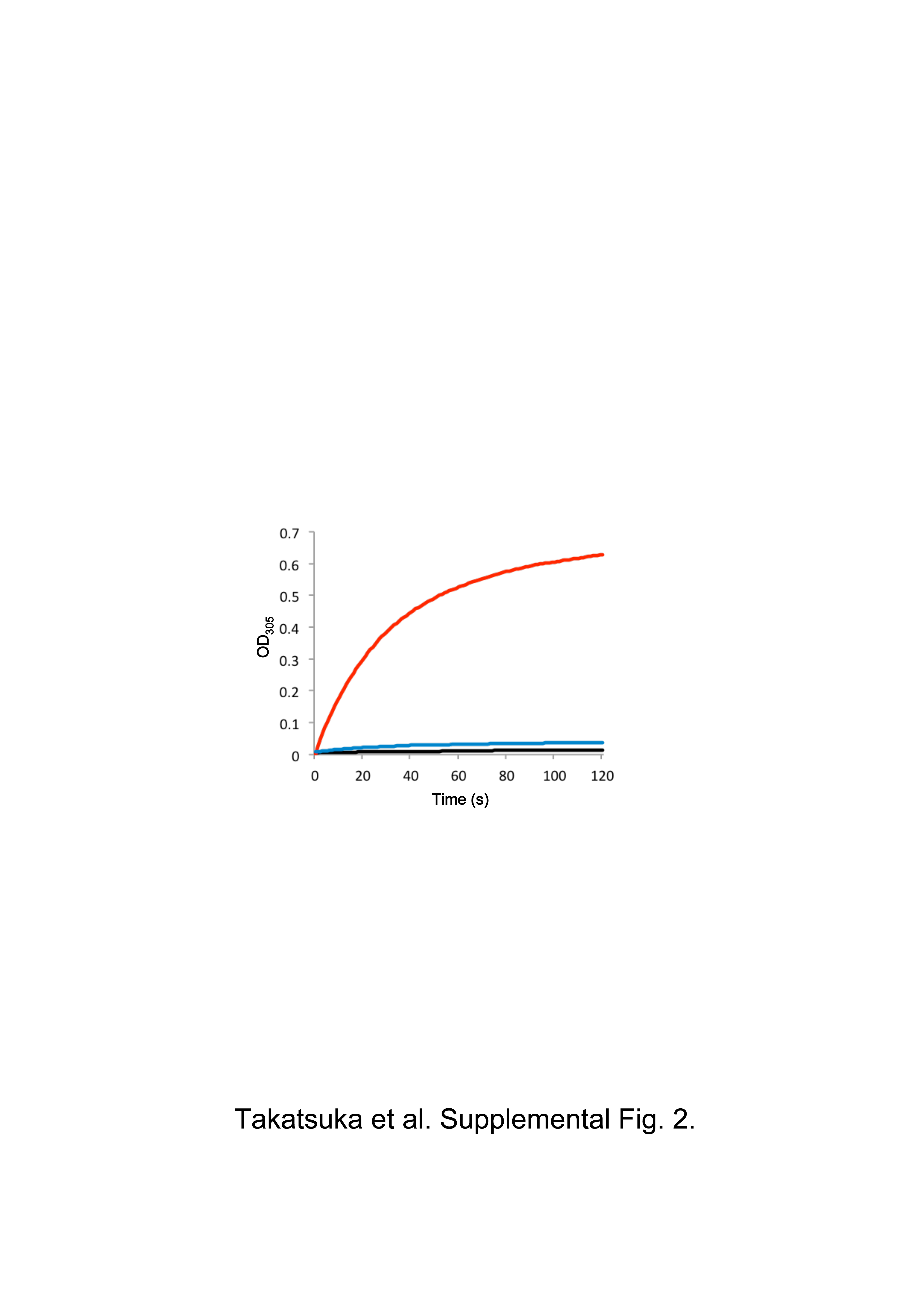

Supplement: Figure S2 — Ferroxidase activity of Ms-MDP1. The conversion from Fe2+ to Fe3+ was scanned by measuring the absorbance at 305 nm. Black line, 0.4 mM FeSO4; blue line, 0.4 mM FeSO4+1.4 µM Histone H1; red line, 0.4 mM FeSO4+1.4 µM Ms-MDP1. (TIF) [file pone.0020985.s002.tif]

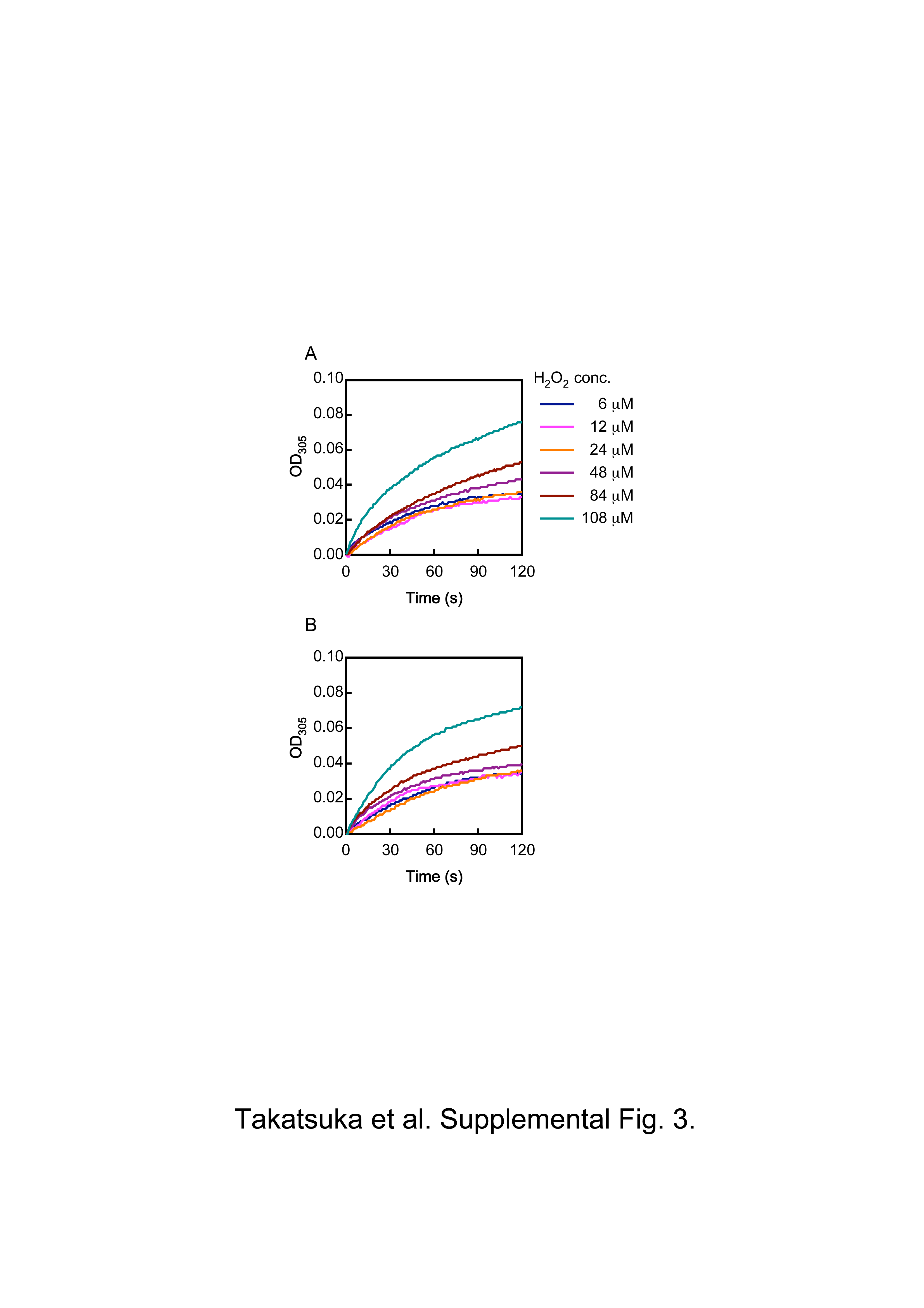

Supplement: Figure S3 — Ferroxidase activity of Mtb-MDP1 and ferritin using hydroxyl peroxide, as the oxidant The conversion from Fe2+ to Fe3+ was monitored by spectral analysis at 305 nm. Hydroxyl peroxide (6–108 mM) were added to the solution of either 0.5 µM Mtb-MDP1 (A) or horse ferritin (B) including with 21 µM FeSO4. (TIF) [file pone.0020985.s003.tif]
